# Disentangling the influence of environmental conditions on sex determination in haplodiploid organisms

**DOI:** 10.1111/1365-2656.70153

**Published:** 2025-10-14

**Authors:** Katharina Wittmann, Alexandra‐Maria Klein, Maximilian Pichler, Michael Staab

**Affiliations:** ^1^ Nature Conservation and Landscape Ecology, Faculty of Environment and Natural Resources University of Freiburg Freiburg Germany; ^2^ Theoretical Ecology University of Regensburg Regensburg Germany; ^3^ Institute of Ecology, Leuphana University Lüneburg Lüneburg Germany

**Keywords:** bees, foraging, landscape, life history, *Osmia cornuta*, population dynamics, resource availability, sex allocation

## Abstract

1. Sex determination is essential for the life history of sexually reproducing organisms. Understanding the mechanism behind sex determination decisions, however, is not trivial, as processes such as random meiosis can shape the sex of the offspring besides environmental conditions. Haplodiploid organisms are relatively unconstrained from these internal influences: Males develop from unfertilized and females from fertilized eggs. Females can thus base sex allocation on their individual parental expenditure and account for the prevalent environmental conditions they live in.
2. We aim to disentangle the influence of environmental conditions on sex determination and resource allocation decisions of haplodiploid organisms (population sex ratio, individual sex allocation probability, individual resource allocation and foraging efficiency).
3. For this, we studied the European orchard bee (*Osmia cornuta*) at a high spatial and temporal resolution in a quantitative field study. We applied a recently developed camera system and deep‐learning‐based evaluation toolset that allowed us to analyse over 1000 pollen collection flights (food provisioning for offspring) and over 800 clay collection flights (nest‐building material) to test whether sex determination and resource allocation in haplodiploid organisms depend on environmental conditions.
4. Contrary to expectations based on established sex determination theories, the overall population sex ratio and individual offspring resource allocation were independent of environmental conditions. Individual sex allocation probability, however, shifted with flower cover, connectivity of seminatural habitat, temperature and progressing season. Pollen and clay collection durations, proxies for foraging efficiency, were not influenced by available resources in the landscape. Instead, pollen collection efficiency decreased with higher temperatures and clay collection efficiency decreased with lower temperatures and progressing season (both represented by increasing flight durations).
5. A short‐term insurance strategy may explain the diverse influences of environmental conditions on individual sex allocation probability, whereas long‐term bet‐hedging might result in consistent offspring resource allocation and population sex ratio within a year, with potential carry‐over effects into the next generation. We therefore conclude that sex determination is not monocausal and that nest‐provisioning females might pursue multiple aims at the same time. We emphasize the importance of long‐term data to further unravel the sex determination mechanisms of sexually reproducing organisms.

## Graphical abstract

The authors unravel how environmental conditions influence sex determination and resource allocation in a haplodiploid bee species. Using high‐resolution field data, they show that individual sex allocation shifts with environmental conditions, while population sex ratio and individual resource allocation remain stable, highlighting the complexity of maternal decision‐making in solitary bees.

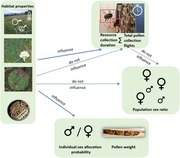

## 1 INTRODUCTION

‘Life history’ describes the survival, growth and reproduction of an organism through its ontogeny. The individual traits of all organisms are optimized via natural selection (optimality theory; Parker & Smith, 1990). However, trade‐offs in energy expenditure are necessary, as resources are always limited in a finite system. For instance, the interplay of intra‐ and interspecific competition and varying local conditions shape the best‐performing actions for each individual, leading to natural variability (Alonzo & Kindsvater, 2008; Parker & Smith, 1990).

An example of a life‐history parameter with a variable optimum is sex determination in sexually reproducing species, which has yet to be completely understood (Bull & Charnov, 1988; Wilson & Hardy, 2009). Based on observations as old as Darwin's (1871), Fisher (1930) postulated a stable 1:1 sex ratio equilibrium of males and females when male and female offspring require equal parental expenditure (Fisher's principle). Depending on the individual life‐history strategy of a species, population sex ratio may thus be influenced by different extrinsic and intrinsic parameters. For instance, sexual size dimorphism is one of the known drivers that shifts the numerical sex ratio towards the smaller and therefore less ‘costly’ sex (optimal sex ratio hypothesis; Torchio & Tepedino, 1980). Considerable variation in population sex ratios, however, is common across species (Stillwell et al., 2010) and has been linked to limited resources (costs of reproduction hypothesis; Myers, 1978) or maternal condition at the time of breeding (Trivers–Willard hypothesis; Trivers & Willard, 1973). Disentangling the influences of the underlying sex determination mechanisms is thus not trivial, albeit essential for species conservation because the long‐term existence of populations is conditional on the occurrence of both sexes at a suitable ratio (Cameron et al., 2017; West & Sheldon, 2002; Wilson & Hardy, 2009).

In haplodiploid species (i.e. females with a diploid chromosome set and males with a haploid chromosome set), mothers have a relatively unconstrained control over the sex of their offspring. Female Hymenoptera, for example, can facultatively contract the spermatheca, which will fertilize the egg and result in female offspring, whereas an unfertilized egg results in male offspring (Gerber & Klostermeyer, 1970). Further, the size of an individual is dependent on the resources that were available at the larval stage and is not determined by genotype (Tepedino et al., 1984). Hymenoptera are therefore suitable organisms to understand essential life‐history traits, including how environmental conditions can alter individual sex and resource allocation decisions of the mother and how they relate to overall population sex ratio (Rehan & Richards, 2010).

As many Hymenoptera are sexually dimorphic, the usually smaller males require as larvae less food provisions than females (Bosch & Vicens, 2002), which lowers individual parental expenditure and shifts the numerical population sex ratio towards males (optimal sex ratio hypothesis; Torchio & Tepedino, 1980). In turn, the population sex ratio is generally female biased when males are larger than females (Sugiura, 1994). Apart from the size of the offspring, the investment costs of the mothers can be influenced by environmental conditions, for example by fluctuations of resources such as mass flowering events (Jauker et al., 2012) due to their sensitivity to habitat changes (IPBES, 2016; Klein et al., 2018; Outhwaite et al., 2022). Female Hymenoptera are thus expected to maximize their individual fitness under varying environmental conditions according to the costs of reproduction hypothesis (Myers, 1978), which postulates the investment in ‘more’ of the relatively ‘cheaper’ male offspring under scarce resources because of lower resource requirements per individual.

This quantitative field study aimed to unravel the underlying mechanisms behind sex determination (population sex ratio and individual sex allocation probability) and individual offspring resource allocation. To address the complexity of the sex determination mechanism in haplodiploid species and to understand the underlying basic concepts, we combined behavioural, population and landscape ecology and used a high temporal and spatial resolution to disentangle the influence of environmental conditions on sex ratio (population scale), sex allocation probability and offspring resource allocation per brood cell (individual scale) of the European orchard bee (*Osmia cornuta*). In contrast to, for example, eusocial honeybees, this cavity‐nesting bee species is solitary, and each female is reproductively active without cooperation among females during reproduction (Bosch & Kemp, 2004).

Quantitative mapping of potential foraging resources does not necessarily reflect the actual resources required by provisioning females (Eckerter et al., 2022; Vaudo et al., 2016), and the true drivers of sex determination and resource allocation may be confounded by unmeasured variables (Cole et al., 2017). Therefore, we assessed individual pollen and clay foraging efficiency, defined as the flight duration required by a female to collect these resources. Pollen is an essential resource for offspring provisioning, and clay for nest building. We additionally assessed pollen flight frequency, meaning the total number of conducted pollen collection flights. A shorter flight duration allows females to collect more resources per unit time, thereby altering parental expenditure, which may influence the population sex ratio (Peterson & Roitberg, 2006). Quantitative documentation of individual resource collection flights, however, is labour‐intensive and inefficient, with usually a human observer being placed next to the bee nests. Available data are thus often restricted to a single locality (Cameron et al., 1996; McKinney & Park, 2012; Richards, 2004; Vicens & Bosch, 2000a), represent few individuals per locality (Bosch & Blas, 1994; Gathmann & Tscharntke, 2002; Visscher & Danforth, 1993) or require many observers at the risk of observation bias (Klein et al., 2004), which makes drawing general conclusions difficult. In our study, we evaluated extensive video material (704 h), comprising over 1000 pollen and over 800 clay collection flights at 20 sites. The recordings were obtained using a recently developed camera system and evaluation toolset (Wittmann et al., 2024), resulting in one of the largest datasets on provisioning behaviour in solitary Hymenoptera to date.

We expected a population sex ratio equilibrium with a higher percentage of males than females due to the smaller size of the males, according to Fisher's principle (Fisher, 1930) and the optimal sex ratio hypothesis (Torchio & Tepedino, 1980). For individual offspring resource allocation, we expected a similar outcome, meaning that the smaller males will receive less provisions than the larger females. We additionally assume a changed individual offspring resource allocation strategy with less pollen provisions for both sexes with decreasing resources (flower cover and pollen provisioning trees in the landscape) according to the costs of reproduction hypothesis (Myers, 1978). In the case of decreasing resource availability, we hypothesize that females would undertake fewer but longer foraging flights, shifting the population sex ratio towards males. Because fluctuating habitat conditions such as temperature can influence the provisioning female during the study period, we hypothesize that a shifted individual sex allocation probability per time towards males can occur with decreasing habitat suitability (costs of reproduction hypothesis; Myers, 1978).

## 2 MATERIALS AND METHODS

### 2.1 Study sites

The study was conducted in the Upper Rhine Valley, in south‐west Germany (47°54′–48°8′ N, 7°37′–7°52′ E). The heterogeneous landscape is mainly used for agriculture, including fruit cultivation and viticulture. Twenty study sites varying in land‐use intensity and resource availability were selected (Figure S1), building upon previous results of *O. cornuta* populations (Wittmann et al., 2023). A study site was defined as an area with a radius of 250 m, which is the average foraging distance of most solitary bees and wasps (Steffan‐Dewenter, 2003; Zurbuchen et al., 2010). The minimum distance between adjacent sites was 1.4 km. The mean annual temperature in the region is 11.8°C, and the mean annual precipitation is 834 mm (period from 1990 to 2022). The study period was April 2021, with a mean temperature of 8.4°C, the coldest April since 1986 (mean temperature April 1990–2022: 11.1°C, Deutscher Wetterdienst, 2024).

### 2.2 Study organism

The European orchard bee (*O. cornuta*) is a cavity‐nesting solitary bee and relevant for pollination in commercial fruit orchards. Each female is responsible for its own offspring. Pollen provisions for the developing offspring are collected in multiple collection flights, and one egg per brood cell is deposited in a linear nest. A female lays on average 8 to 10 eggs during her lifespan (ranging from 1 to 23; Bosch & Vicens, 2006). Each finalized brood cell is sealed with a plug made of clay, also being collected in several flights (Bosch, 1994). Females are larger than males, and provisions for male offspring are typically smaller than provisions for female offspring (Wittmann et al., 2023).

### 2.3 Data collection

A standardized trap nest (10.5 cm diameter) for cavity‐nesting solitary Hymenoptera was placed at the centre of each study site and contained reed internodes of 20 cm length, with diameters between 8 and 11 mm (Krombein, 1967; Staab et al., 2018). To observe the bees simultaneously on all sites, a camera system comprising a single‐board computer and a camera (Raspberry Pi, Raspberry Pi Foundation, Cambridge, UK) was installed in front of one opening (detailed description of camera system and evaluation toolset: Wittmann et al., 2024). All traps were installed at the end of March and scheduled recordings were done from 10 April 2021 to 29 April 2021. The system recorded European orchard bees flying in and out of the trap nest from 10:00 to 11:00 and from 15:00 to 16:00 CEST, two of their main flight times (Vicens & Bosch, 2000b). Fifteen male and 15 female cocoons of commercially bred *O. cornuta* from a nearby locality (WAB Mauerbienenzucht, Konstanz, Germany) were placed per trap to ensure the presence of adult individuals of both sexes and to supplement local populations.

During the sampling period, the videos from the two recording times per day and all completed nests (identifiable by the characteristic plug made of clay) were collected each week (three times; coded as ‘collection day’). Removed reed internodes were replaced with internodes of a similar diameter. The collected nests were opened, and internode diameter and pollen weight of each brood cell (excluding the egg) were documented with a precision scale (to 0.0001 g). Offspring and their corresponding resources were afterwards reared separately in 48‐well plates (Tissues Culture Plates, VWR, Darmstadt, Germany) to link each developing individual to individually provided resources (Becker & Keller, 2016). The openings of the well plates were sealed with adjusted Ceaprene stoppers (22 mm, Greiner‐Bio‐One GmbH, Frickenhausen, Germany). After storage at ambient temperature, the dormant period was simulated from mid‐November 2021 until mid‐March 2022 in a refrigerator at 2.8°C. The individuals were sexed after hatching in spring 2022.

### 2.4 Video analysis

Video sequences containing bees were filtered with an adapted evaluation toolset (introduced by Wittmann et al., 2024) via a convolutional neural network (CNN) (YOLOv5 v.7.0; Jocher et al., 2022). Since the original publication, the toolset was improved by adding more training images to further enhance detection accuracy. The CNN was trained with four NVIDIA RTX 2080 Ti GPUs (NVIDIA, Santa Clara, USA) and default hyperparameters for 300 epochs, until detection accuracy did not significantly improve (optimum at 298 epochs). The final model had a true positive ratio of 0.99 (to 0.01 false‐negative ratio) and a false‐positive ratio of 1.00 (for more details on the model, see figshare repository, Wittmann et al., 2025).

After having filtered the recorded original bee videos that comprised 2186 GB data and 704‐h material, the remaining video material (48 h) was evaluated by one human observer who recorded individual flight durations by determining the time a bee was leaving a specific reed internode and the same bee entering the reed internode again. In case the bee carried clay or deposited pollen (Figure S2), the foraging efficiency, meaning the time a bee required to collect pollen or clay (‘pollen collection duration’ or ‘clay collection duration’) was considered for analysis. In case of uncertainty, the more detailed unfiltered videos were additionally referenced to determine flight durations of individual bees. Afterwards, pollen flight frequency, that is the sum of all conducted pollen collection flights per site and per collection day, was calculated.

### 2.5 Environmental conditions

We assessed different habitat properties representing potential foraging and provisioning resources, including herbaceous flower cover, pollen provision trees and connectivity of seminatural habitat. We also added temperature as an external parameter. Further, seasonal progression was considered to account for short‐term changes in conditions during the observation period.

Temperature was recorded at the start, mid and end of each 1‐h video via temperature loggers (HOBO Pendant, 64 K, Onset, Bourne, USA), with the median used in analyses. Four sites had partially missing values due to malfunctioning temperature loggers (14% total). These missing values were predicted site‐specific from the data of the most correlated site via linear regression (*R* ^2^ > 0.92).

Flower cover was assessed at 21 subplots per site (each 1 m^2^) by taking an overhead photograph with a digital camera at each subplot (between 27 April 2021 and 29 April 2021). The subplots were evenly spread at a distance of 0, 25, 50, 100, 175 and 250 m away from the site centre, towards the four cardinal directions (avoiding forests and hedges). For each photograph, the percentage of flowers from herbaceous plants that are relevant for *O. cornuta* (following Westrich, 2019; Table S1) was estimated by using a virtual grid with one square representing 1%.

In addition to herbaceous plants, *O. cornuta* in the study area especially forages for pollen from Rosaceae trees and *Salix* species (Eckerter et al., 2022). These trees, which all flowered during the study period, were combined in the variable ‘pollen provision trees’ (Table S2) and comprise the percentage of combined crown area of such trees per site.

Seminatural habitat connectivity, which is often related to habitat suitability and abundance of wild bees (Uzman et al., 2020), was represented by the largest patch of seminatural habitat per site in percent. The variable comprises the area of extensively managed meadows, gardens, woody structures (excluding intensive commercial fruit orchards), fallows and unpaved paths. The variable was taken from a different study from the previous year (2020) that was conducted on the same sites (Wittmann et al., 2023).

A detailed overview of all environmental variables and site‐specific parameters is given in Tables S3 and S4 (complete dataset available at Wittmann et al., 2025).

### 2.6 Statistical analyses

All statistical analyses were conducted using R (4.4.1, R Core Team, 2024). Numerical fixed effects were centred and scaled (mean = 0, SD = 1) to get standardized effect estimates.

We used population sex ratio, which was calculated by dividing the number of hatched females by the total number of hatched individuals per site (sex ratio = *F*/(*F* + *M*), Wilson & Hardy, 2009) as response variable and tested for the influence of resource availability (flower cover, pollen provision trees and seminatural habitat connectivity), pollen flight frequency (log‐transformed to improve variance homogeneity) and temperature using a generalized linear model (GLM; packages lme4, Bates et al., 2015 and lmerTest, Kuznetsova et al., 2017) with binomial error distribution (Table S5).

As environmental conditions in spring can quickly change, we tested individual sex allocation probability (the probability of producing a female offspring per brood cell) for relationships with resource availability variables, temperature and collection day. For this binary analysis, brood cells developed into females were coded as 1 and brood cells developed into males as 0. Consequently, the analysis assessed the relationship between the likelihood of a cell being female and the environmental conditions. We also included the internode diameter of the respective nest as a fixed effect, with smaller diameters typically associated with a higher probability of male offspring hatching (Longair, 1981). With these variables, we calculated a GLM with binomial error distribution (Table S5). In this model, no random intercept to account for the hierarchical structure of the data was fitted, as it explained no variance (Table S6).

Individual resource allocation allows an assessment of the size of the future offspring. We therefore tested the relationship of individually allocated resources (pollen weight in mg; individual scale) with the same fixed effects (resource availability variables, temperature and collection day). Individual reed internode nested in study site was added as a random intercept in the linear mixed‐effects model (LMM), because one mother generally builds all brood cells in one reed until completion of the nest. Hence, this random intercept also accounts for eventual size differences among mother bees. The sex of the hatched offspring was included as a fixed effect to account for the sexual dimorphism of the target species (Table S5). Flower cover and pollen provisioning trees were measured only once at the end of the field season, which could have led to a potential bias. However, results did not change when restricting the analysis to data from the third collection day, suggesting that this limitation did not substantially affect the findings (Table 1; Table S7).

**TABLE 1:** Numerical results of the models that tested for influences on sex allocation probability (the probability of producing female offspring per brood cell) (binomial GLMM), pollen weight (linear LMM), and foraging efficiency (meaning pollen and clay collection flight duration; negative binomial GLMMs) at the individual scale.

Response	Fixed effect	Estimate ±SE	*z*−/*t*‐value	*p*‐value
Sex allocation probability [brood cells developed into females = 1, brood cells developed into males = 0]	**Collection day**	**−0.525 ± 0.153**	**−3.437**	**<0.001**
	**Flower cover**	**0.434 ± 0.147**	**2.947**	**0.003**
	**Temperature**	**0.452 ± 0.164**	**2.748**	**0.006**
	**Seminat. habitat connectivity**	**−0.325 ± 0.138**	**−2.352**	**0.019**
	Internode diameter	0.121 ± 0.117	1.033	0.302
	Pollen provision trees	−0.045 ± 0.122	−0.371	0.711
Pollen weight [mg]	**Sex (male or female)**	**153.495 ± 5.804**	**26.445**	**<0.001**
	**Internode diameter**	**15.035 ± 3.961**	**3.796**	**<0.001**
	Collection day	−9.946 ± 5.420	−1.835	0.072
	Flower cover	8.980 ± 7.548	1.190	0.252
	Seminat. habitat connectivity	−6.764 ± 8.735	−0.774	0.455
	Pollen provision trees	−3.934 ± 6.516	−0.604	0.557
	Temperature	−3.016 ± 6.817	−0.442	0.660
Pollen collection duration [s]	**Recording day**	**0.146 ± 0.023**	**6.430**	**<0.001**
	**Temperature**	**0.061 ± 0.023**	**2.610**	**0.009**
	Flower cover	−0.027 ± 0.033	−0.810	0.419
	Seminat. habitat connectivity	−0.029 ± 0.041	−0.700	0.481
	Pollen provision trees	−0.002 ± 0.033	−0.050	0.962
Clay collection duration [s]	**Temperature**	**−0.102 ± 0.046**	**−2.210**	**0.027**
	Seminat. habitat connectivity	0.129 ± 0.090	1.440	0.149
	Flower cover	−0.094 ± 0.082	−1.150	0.251
	Recording day	0.049 ± 0.049	1.010	0.314
	Pollen provision trees	0.036 ± 0.077	0.470	0.637

*Note*: Standardized model estimates (*±*SE), *z*−/*t*‐values, and *p*‐values are shown. Significant relationships (*p* < 0.05) are indicated in bold. Variables are each sorted by decreasing *z*−/*t*‐values. Model results for population sex ratio can be found in Table S9 and do not contain any significant relationships.

To gain more information about the time a female requires to gather resources in the landscape, we tested the pollen and clay collection duration (i.e. foraging efficiency; individual scale) for relationships with resource availability variables, seasonal progression (day of the recording) and temperature (Table S5). A negative binomial generalized linear mixed‐effects model (GLMM) with a random intercept (individual reed internode nested in study site) was applied to mitigate overdispersion (package glmmTMB 1.1.9; Brooks et al., 2017).

The models described directly above test for direct relationships between response variables and environmental conditions. As relationships among population sex ratio, offspring resource allocation, and environmental conditions are likely not only direct but may also be mediated indirectly, we used a confirmatory path analysis (sensu Shipley, 2009; package piecewiseSEM 2.3.0.1; Lefcheck, 2016). Our aim was to determine whether the influence of environmental conditions (temperature, flower cover and pollen provision trees) on population sex ratio was direct or mediated by pollen flight frequency. The a priori model structure was informed by the outcome of the previous analyses and by known relationships, using only observed data and not latent variables. For example, a lower overall pollen flight frequency may indicate prolonged collection flight durations due to, for example, a lower resource availability (costs of reproduction hypothesis, Myers, 1978) or declining health of the provisioning mother (Trivers–Willard hypothesis; Trivers & Willard, 1973). As seminatural habitat connectivity might be mediated by the availability of different resources, a path from seminatural habitat connectivity to flower cover and to pollen provision trees was included (Cole et al., 2017; Eeraerts et al., 2021). To account for the spring weather at the time of the study with an expected increasing temperature over time, we added a partial and nondirectional correlative error term between temperature and seasonal progression. Because the progressing season can also have a direct effect on sex ratio by shifting towards the less costly male offspring (e.g. due to the age of the mother), we further included a direct path from seasonal progression to sex ratio in the path model. The component models of the path model used the same data as the GL(M)Ms above (component model structure: Table S6; parameters for the path model: Table S8), including the same random effects structure to account for the hierarchical structure in the data. Overall model fit of this a priori model, which tests for the conditional independence of the assumed paths, was evaluated with a d‐separation test (Shipley, 2009).

Residuals of all GL(M)Ms and of the component models in the path analysis were checked for overdispersion, heteroscedasticity, zero‐inflation and outliers (package DHARMa, 0.4.6; Hartig, 2021), which were not apparent. Residuals of L(M)Ms were normally distributed. All models (including component models in the path analysis) were tested for multicollinearity via variance inflation factors (always <3, i.e. collinearity unlikely) (package performance 0.12.2; Nakagawa et al., 2017) and spatial autocorrelation (Moran's *I*; *p* > 0.05). A quadratic term for temperature did not improve the fit of any model. We also tested whether cell position influenced the probability of offspring survival with a GLMM with binomial error distribution (coded: 1 = survival or 0 = dead larva/egg), the fixed effect cell position (1 = outermost cell, increasing count inwards) and a random intercept (reed internode nested in site).

## 3 RESULTS

We evaluated video material from 20 sites, which was created by a recently introduced camera system and evaluation toolset (Wittmann et al., 2024). The videos comprised 3960 flights of *O. cornuta* in and out of their nest, from which 1011 were pollen collection flights (17 sites; median duration = 1060 s ± 634 s SD) and 873 clay collection flights (17 sites; median duration = 123 s ± 257 s SD). A total of 84 reed internodes from 17 sites were collected. The individually provisioned resources were weighted for 450 cells, from which 344 offspring hatched that were sexed (with no influence of cell position within a reed internode on mortality, *z* = −1.7, *p* = 0.09).

**FIGURE 1:**
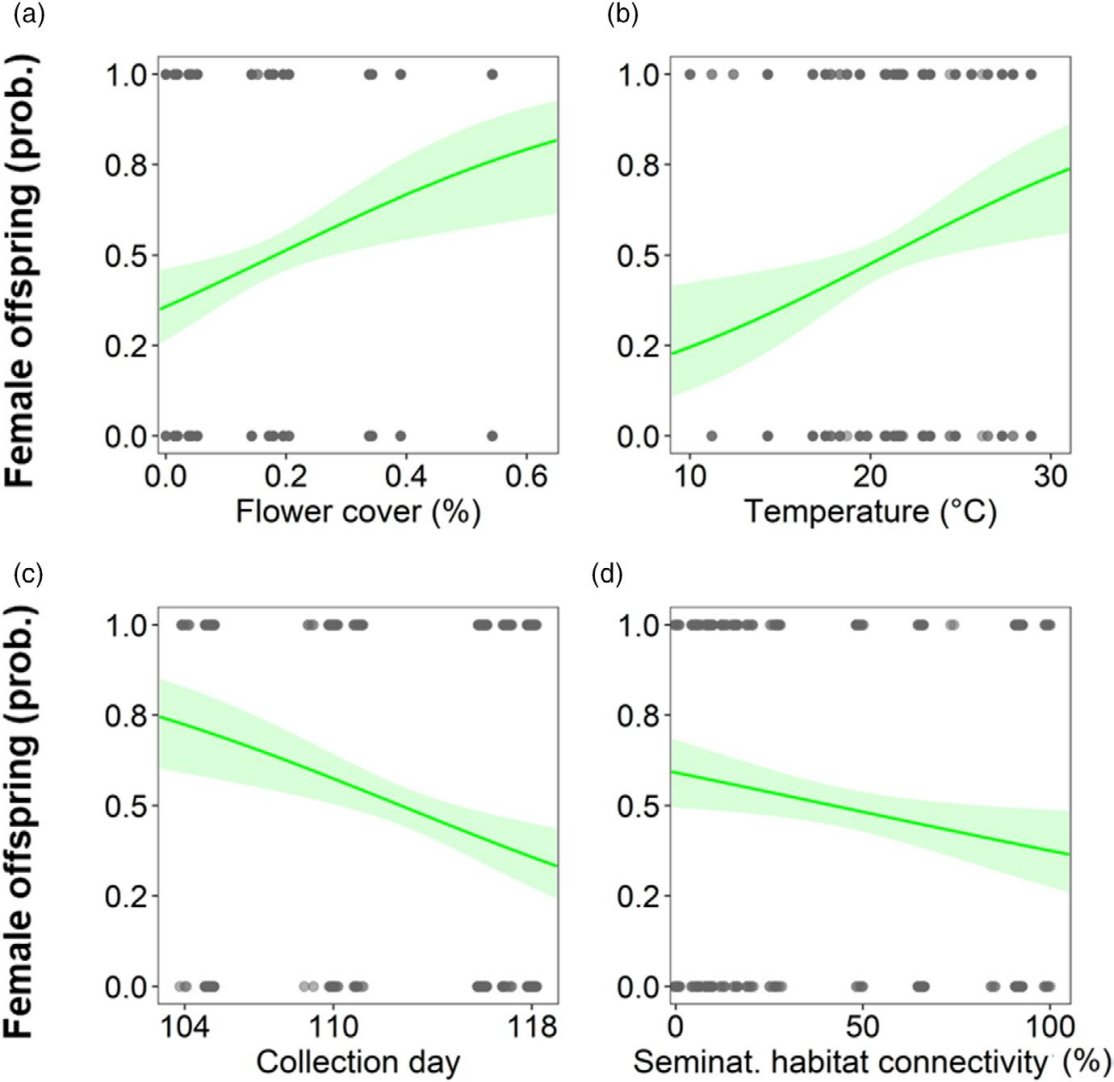
Visualization of model results (marginal effects) depicting the relationship of individual sex allocation probability and flower cover, temperature, collection day and seminatural (seminat.) habitat connectivity (only fixed effects with *p* < 0.05). Brood cells that developed into females were coded as 1 and brood cells that developed into males as 0. The probability to produce a female offspring increased with (a) more flower cover (*z* = 2.947, *p* = 0.003) and (b) higher temperature (*z* = 2.748, *p* = 0.006), and decreased with (c) progressing season (represented by collection day; *z* = −3.437, *p* < 0.001) and (d) seminatural habitat connectivity (*z* = −2.352, *p* = 0.019). Solid lines indicate model predictions with 95% confidence intervals, which are shown as shaded polygons. Full model results are shown in Table 1.

Against initial predictions, population sex ratio was not influenced by any tested variable (Table S9; sex ratio median = 0.5 ± 0.2 SD). Individual sex allocation probability, however, was related to seasonal progression (represented by collection day), flower cover, temperature and seminatural habitat connectivity (Table 1). The probability for female offspring increased with higher flower cover (*z* = 2.947, *p* = 0.003; Figure 1a) and at higher temperatures (*z* = 2.748, *p* = 0.006; Figure 1b). Furthermore, a female offspring became less likely later in spring with increasing collection day (*z* = −3.437, *p* < 0.001; Figure 1c) and increasing seminatural habitat connectivity (*z* = −2.352, *p* = 0.019; Figure 1d). Individual offspring resource allocation, represented by provisioned pollen weight, was only influenced by internode diameter and sex. Pollen weight increased in internodes with larger diameter (*t* = 15.035, *p* < 0.001; Table 1) and for brood cells that developed into females (*t* = 153.495, *p* < 0.001) (Figure S3).

**FIGURE 2:**
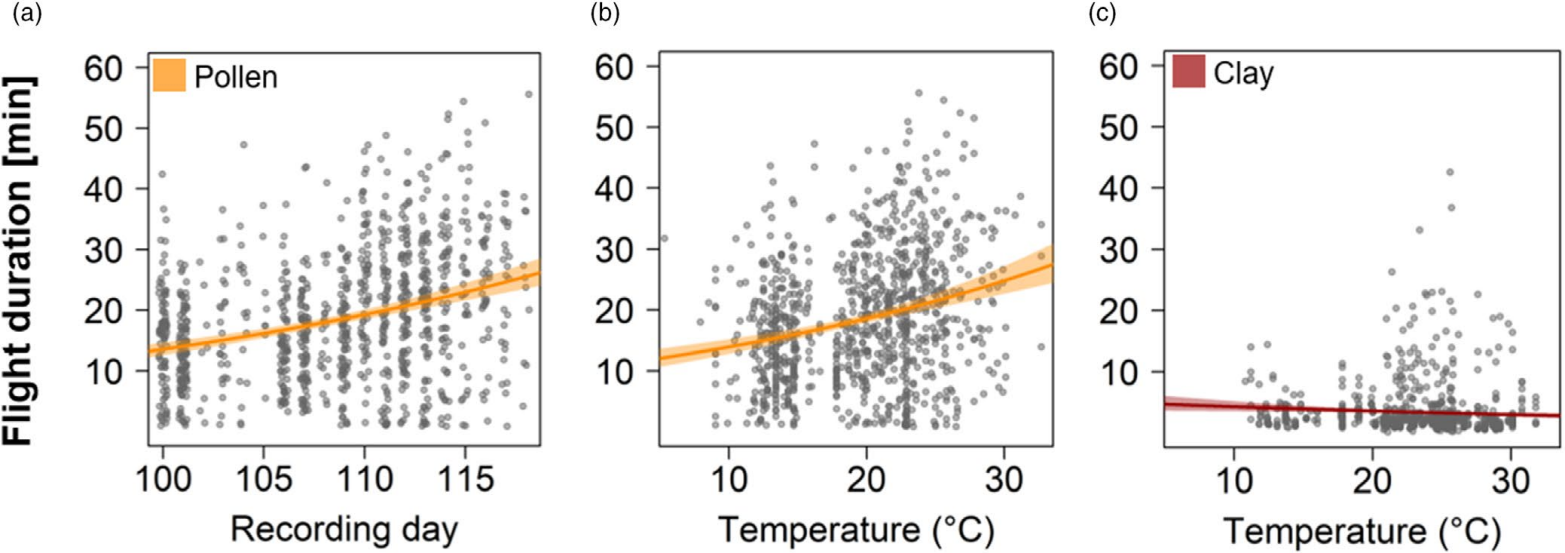
Visualization of model results (marginal effects) depicting the influence of recording day and temperature on pollen and clay foraging efficiency (only fixed effects with *p* < 0.05). Pollen collection flight duration was longer (a) later in the season (*z* = 6.430, *p* < 0.001) and (b) at higher temperatures (*z* = 2.610, *p* = 0.009). Clay was collected faster at (c) higher temperatures (*t* = −2.210, *p* = 0.027). Solid lines indicate model predictions with 95% confidence intervals, which are shown as shaded polygons. Please note that the visualization of the model results uses min for an easier interpretation while statistical analyses were conducted in s. Full model results are shown in Table 1.

Pollen foraging efficiency was related to progressing season, represented by recording day, and temperature (Table 1). Bees foraged for a longer time to gather pollen later in the season (*z* = 6.430, *p* < 0.001; Figure 2a) and at a higher temperature (*z* = 2.610, *p* = 0.009; Figure 2b). Clay foraging efficiency was only related to temperature (Table 1), with a decrease in collection time with increasing temperature (*t* = −2.210, *p* = 0.027; Figure 2c).

**FIGURE 3:**
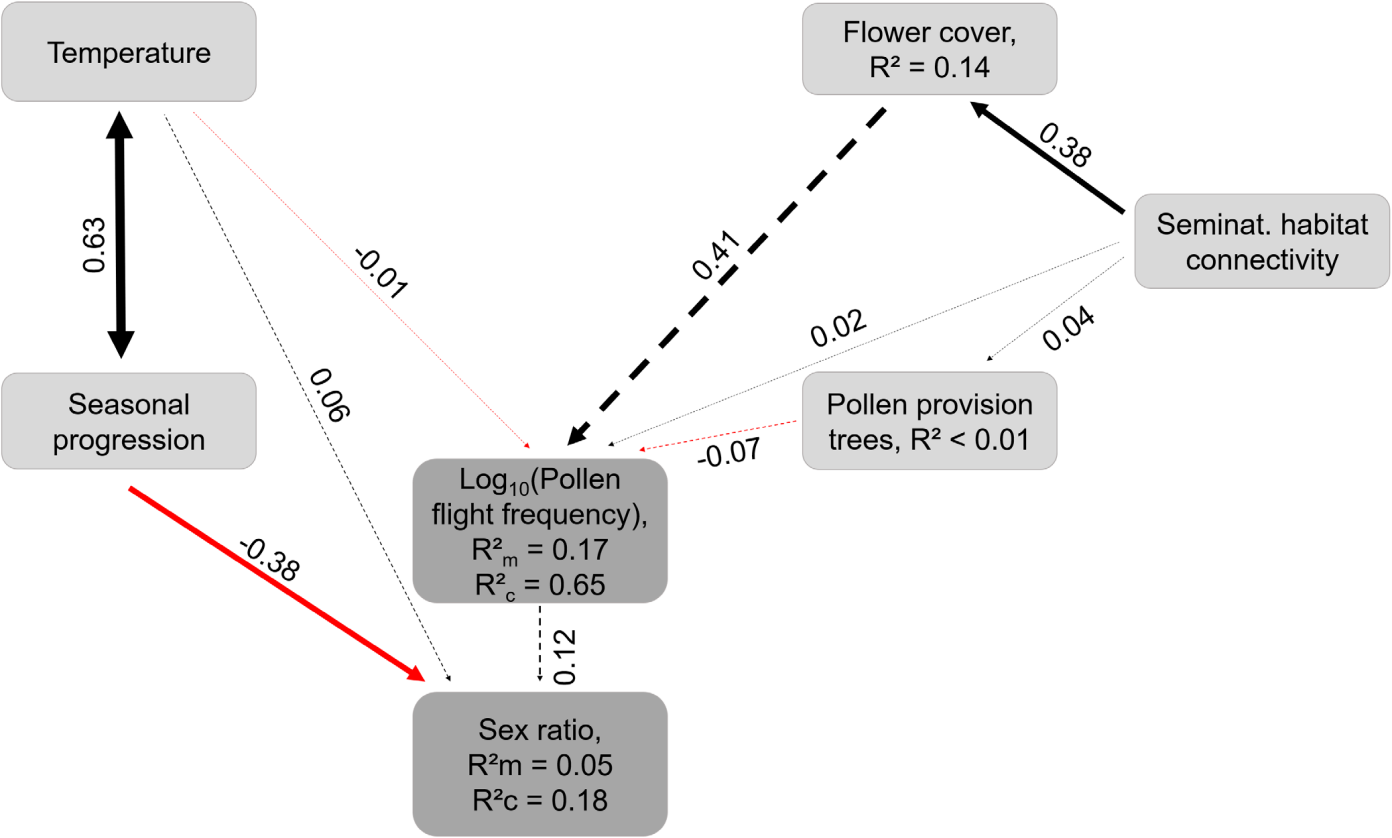
Confirmatory path model illustrating that resource availability did neither influence the total number of pollen flights (log‐transformed) nor population sex ratio per site (Fisher's *C* = 15.4, *p* = 0.64, indicating high statistical support). Seasonal progression, however, shifted population sex ratio towards males. A red continuous arrow represents a significant negative and a black continuous arrow a significant positive relationship (*p* < 0.05). Dashed lines represent nonsignificant paths. Values alongside arrows give standardized path coefficients. *R* ^2^m (marginal *R*‐squared) represents the proportion of variance that is explained by the fixed effects in the model, while *R* ^2^c (conditional *R*‐squared) represents the proportion of variance that is explained by both fixed and random effects. Component models are described in Table S6 and full details on the path model are available in Table S8.

The path model received high statistical support (Fisher's *C* = 15.4, df = 18, *p* = 0.64). However, no direct or indirect influences of environmental conditions on population sex ratio or pollen flight frequency were found, apart from a positive (albeit nonsignificant) trend of seminatural habitat connectivity, which was mediated by flower cover (standardized path coefficient 0.41) (Figure 3). Nevertheless, seasonal progression, which was correlated with temperature (standardized path coefficient 0.63), changed the sex ratio towards an increased number of males (standardized path coefficient −0.38). Overall, the hypothesized relationships with exogenous variables did not explain sex ratio (*R* ^2^m = 0.05 and *R* ^2^c = 0.18) and total pollen flights (*R* ^2^m = 0.17 and *R* ^2^c = 0.65) well (Table S8).

## 4 DISCUSSION

Overall, none of the environmental variables consistently explained variation in population sex ratios across sites in the haplodiploid species *O. cornuta*. However, Fisher's principle (Fisher, 1930), which predicts a balanced population sex ratio based on parental investment in male and female offspring, was partially supported, as individual sex allocation reflected the multifaceted maternal investment strategies that are necessary under changing environmental conditions (Rosenheim et al., 1996). In our study, the sex of each brood cell in our target species (i.e. the individual sex allocation probability) was adjusted depending on seminatural habitat connectivity, flower cover and temperature, which can all influence the body condition of the mother at the time of brood cell provisioning. Thus, heterogeneity in environmental conditions over time (also indicated by the changing population sex ratio with progressing season) most likely requires an adaptation of provisioning strategies (Wong & Forrest, 2021) to optimize energy expenditure of the mother and to produce the optimal population sex ratio under the available environmental conditions, as proposed by the evolutionary stable strategy (Parker & Smith, 1990).

Offspring resource allocation is equally vital for the future performance of each individual, as it can determine survival and future reproductive success (Boggs, 1992). While the sex in Hymenoptera is determined by haplodiploidy, adult body size is strongly correlated with the amount of food a larva consumes (Bosch & Vicens, 2002; Tepedino et al., 1984). A larger size has been linked to increased offspring fecundity and overwintering survival (Bosch & Kemp, 2004; Honěk, 1993). Although larger offspring require more parental expenditure, pollen weight in our data was independent of available resources and depended solely on sex and internode diameter, both important indicators of offspring body size (Roulston & Cane, 2000). These findings point towards the optimal allocation theory (Smith & Fretwell, 1974), as the individual resource allocation appears to follow an optimized provisioning strategy for each offspring (compare Bosch, 1994), rather than being adjusted in response to environmental conditions (conditional sex allocation, Charnov, 1982; see also Seidelmann et al., 2010).

To further disentangle the influence of resource availability and parental expenditure for provisioning and nest building, we evaluated almost 4000 individual flights (including 1011 pollen and 873 clay collection flights) with the aid of a newly developed camera system and evaluation toolset (Wittmann et al., 2024). Interestingly, pollen foraging efficiency (represented by pollen collection duration) and pollen flight frequency per time were not influenced by any variable related to resources in the landscape. As provisioned pollen by *O. cornuta* is not limited to woody plant species such as *Rosaceae* and *Salix* taxa (Eckerter et al., 2022) but also contains herbaceous plants, the result likely derives from an adaptation of provisioning strategies depending on the most limiting factor at the moment of the foraging flight. Specifically, the atypically cold spring in 2021 and the cold period between the 12th and 19th April (mean temperature = 8.4°C) in the middle of the study period might have negatively affected pollen production of fruit trees (Faust, 1989) despite consistent blooming during the observation period. Even though *O. cornuta* is a generalist species, seminatural habitats are preferred for pollen collection, which not only increases intraspecific competition for pollen in such suitable habitat (Diekötter et al., 2014) but also can lead to a higher parasitism pressure (Coudrain et al., 2014; Eckerter et al., 2022), because individuals are clustered in few seminatural habitat patches. According to the Trivers–Willard hypothesis (Trivers & Willard, 1973), a decline in the health of a provisioning mother over time may shift the offspring sex ratio towards the sex with lower variance in reproductive success. In the case of *O. cornuta*, however, this hypothesis would predict an increase in female offspring because females typically invest more time and resources into provisioning and constructing their brood cells. Albeit the assumed mechanism is speculative, this restriction limits the number of offspring they can produce, resulting in lower variance in reproductive success. By contrast, males may have a higher potential for reproductive success, but they also experience higher variance due to competition for mating opportunities (Trivers & Willard, 1973). However, no increase in female offspring was observed, making this scenario unlikely. Adversely, the cold and rainy weather conditions during the study period might also have reduced interspecific competition. Due to its high endothermic warm‐up rate, *O. cornuta* is able to fly at lower temperatures and worse weather conditions, including windy and rainy days, than, for example, honeybees and most other solitary bees (Bosch & Blas, 1994; Monzón et al., 2004; Vicens & Bosch, 2000b). *Osmia cornuta* females might have undertaken longer pollen collection flights with higher temperatures to combine foraging for nectar for themselves, collecting pollen provisions and scouting for suitable pollen patches at the same time. Because larvae and adults alike require specific amino acids, carbohydrates and proteins that directly relate to their health (Murawska et al., 2022; Persson et al., 2018; Vaudo et al., 2016), the identification of less preferred but complementary pollen and nectar sources is vital to complement nutritional requirements and the diet of the offspring, especially under suboptimal conditions (Filipiak et al., 2022; Persson et al., 2018). Our findings are thus consistent with the costs of reproduction hypothesis (Myers, 1978), as indicated by a shift towards a more male‐biased sex ratio with progressing season and a nonsignificant trend of seminatural habitat connectivity mediated by flower cover that affected the number of pollen collection flights. The results suggest that provisioning females, experiencing progressive depletion of body reserves, may have increasingly prioritized a less energetically costly investment strategy, resulting in the production of more male offspring (compare Ito, 2019).

Resource collection not only incorporates pollen collection but also collecting clay as building material to seal a brood cell or close the nest. The partitions between cells protect offspring from predation or parasitism (Du et al., 2025). Thus, foraging for nest‐building material is a vital part of the life history of non‐parasitic solitary bees and wasps that is severely understudied and deserves more research. In our study, we collected the largest dataset of individual nesting material collection times to date (e.g. Bosch & Blas, 1994; Levin, 1966). In contrast to pollen foraging efficiency, warmer temperatures increased clay foraging efficiency (represented by a shorter clay collection duration). *Osmia* have been observed to gather clay from any suitable source in the surroundings of their nests before, coming back to the same spot and collecting adjacent to each other (Bosch, 1994; Levin, 1966). An increased efficiency is thus expected because clay is heavier than pollen and flying in cold conditions is less efficient (Heinrich, 1974). There are Hymenoptera, however, that require a more specific source for their nesting partitioning walls, which can influence decisions when foraging for building material. For instance, the pebble bee *Dianthidium ulkei* requires resin as nest‐building material, which is spatially less predictable than clay (Frohlich & Parker, 1985).

In summary, the variation in population sex ratio in our field study could not be explained by environmental conditions, including resource availability. This result was unexpected, as studies suggested a consistent shift in numerical sex ratio towards males in *O. cornuta* (Bosch, 1994; Bosch & Vicens, 2006). Nevertheless, individual sex allocation probability was influenced by environmental parameters such as temperature and seminatural habitat connectivity in this study, which was not found in the same area the previous year (Wittmann et al., 2023). Hence, we hypothesize that responses of the sex determination mechanism to environmental conditions involve not only short‐term adaptations to mitigate challenging conditions at the time of nest construction (costs of reproduction hypothesis, Myers, 1978) but also long‐term strategies to balance eventually unfavourable population sex ratios in subsequent years (long‐term bet‐hedging) (Haaland et al., 2019). We base this hypothesis on other studies that record a fluctuating population sex ratio in other haplodiploid species between years (e.g. *Osmia lignaria*, Tepedino & Torchio, 1982; *Auplopus militaris*, Zanette et al., 2004).

Although we conducted one of the most comprehensive studies about sex determination and offspring resource allocation with high temporal and spatial resolution, minor fluctuations of pollen foraging efficiency may not have been detected, as *O. cornuta* can be active for more than 8 hours per day (Vicens & Bosch, 2000b) and for logistical reasons, we only recorded 2 h per day (from 10:00 to 11:00 and from 15:00 to 16:00). Further long‐term investigations could therefore include more sites, a more detailed measure of resource availability and more abiotic parameters such as rain or wind (Brittain et al., 2013; Switanek et al., 2017). A longer time window for recordings from more geographic regions could also be considered. Because changes in body size of offspring and clutch size have the potential to alter sex determination and resource allocation decisions across generations (Bosch, 2008; Pardee et al., 2022), they could be included to refine further studies as well. In short, there are still many promising topics for future research, including the need for more long‐term data across generations and the need for more in‐depth quantitative field data from different organisms and across subsequent years (Breda et al., 2022). This demand has already been called for by Tepedino and Torchio (1982) over 40 years ago. We also emphasize the need to overcome reporting bias (Festa‐Bianchet, 1996) to fully disentangle the sex determination mechanism of sexually reproducing species.

## 5 CONCLUSION

This study provided evidence that individual sex allocation of a haplodiploid organism is reflecting the current local conditions a provisioning female is facing. Overall population sex ratio, however, was not directly influenced by environmental conditions including resource availability. We conclude that site‐dependent population sex ratios are common, although maintaining a stable equilibrium between male and female offspring is a vital life‐history trait of sexually reproducing organisms to ensure the success of future generations. We thus presume underlying long‐term (bet‐hedging) and short‐term (mitigation of current conditions) strategies as essential life‐history parameters that offer potential for further research.

## AUTHOR CONTRIBUTIONS

Katharina Wittmann and Michael Staab conceived the idea; Katharina Wittmann designed the study, collected data and analysed it with the support of Michael Staab and Maximilian Pichler. Katharina Wittmann wrote the manuscript with input from all authors who gave approval for publication.

## FUNDING INFORMATION

Michael Staab is indebted to the Baden‐Württemberg Foundation for the financial support of this research project by the Baden‐Württemberg Foundation (grant number 1.16101.19). We acknowledge support by the Open Access Publication Fund of the University of Freiburg and the Wissenschaftliche Gesellschaft Freiburg.

## CONFLICT OF INTEREST STATEMENT

The authors declare that they have no conflict of interest.

## Supporting information


**Table S1.** Identified flowering herbaceous plant species on study sites.
**Table S2.** Identified bee‐relevant trees/shrubs.
**Table S3.** Summary of environmental variables that describe the 20 study sites.
**Table S4.** Overview of hatched offspring, sex ratio per site, total number of flights (including pollen and clay collection flights) and environmental predictors.
**Table S5.** Model structure of all generalized linear (mixed‐effects) models and linear mixed‐effects models.
**Table S6.** Component models for the path model.
**Table S7.** Model results of pollen weight allocation in the third collection period only.
**Table S8.** Parameters of the path model.
**Table S9.** Model results of sex ratio per site.
**Figure S1.** Overview of the 20 study sites in the surroundings of Freiburg, Germany.
**Figure S2.** Representative images of bees transporting (a) pollen on its scopa and (b) clay in its mandibles as nesting materials.
**Figure S3.** Visualization of model results (only predictors with *p* < 0.05) for the influence of sex and internode diameter on pollen weight.

## Data Availability

Dataset, R script and YOLOv5 training data including training weights are available in figshare: https://doi.org/10.6084/m9.figshare.27108733 (Wittmann et al., 2025).
