# Supplementary material for: Disentangling the influence of environmental conditions on sex determination in haplodiploid organisms
Source: J Anim Ecol. 2025 Oct 14;94(12):2584–96. doi: 10.1111/1365-2656.70153 (PMC12673240; doi:10.1111/1365-2656.70153)
Supplement: Supplementary file 1 — Table S1. Identified flowering herbaceous plant species on study sites. Table S2. Identified bee‐relevant trees/shrubs. Table S3. Summary of environmental variables that describe the 20 study sites. Table S4. Overview of hatched offspring, sex ratio per site, total number of flights (including pollen and clay collection flights) and environmental predictors. Table S5. Model structure of all generalized linear (mixed‐effects) models and linear mixed‐effects models. Table S6. Component models for the path model. Table S7. Model results of pollen weight allocation in the third collection period only. Table S8. Parameters of the path model. Table S9. Model results of sex ratio per site. Figure S1. Overview of the 20 study sites in the surroundings of Freiburg, Germany. Figure S2. Representative images of bees transporting (a) pollen on its scopa and (b) clay in its mandibles as nesting materials. Figure S3. Visualization of model results (only predictors with p < 0.05) for the influence of sex and internode diameter on pollen weight. [file JANE-94-2584-s001.pdf]

Supporting information to *Disentangling the influence of environmental conditions on sex determination in haplodiploid organisms*

Tables **2**

Table S1. Identified flowering herbaceous plant species on study sites 2

Table S2. Identified bee-relevant trees/shrubs 3

Table S3. Summary of environmental variables that describe the 20 study sites 4

Table S4. Overview of hatched offspring, sex ratio per site, total number of flights (including pollen and clay collection flights) and environmental predictors 5

Table S5. Model structure of all generalized linear (mixed-effects) models and linear mixed-effects models. 6

Table S6. Component models for the path model. 7

Table S7. Model results of pollen weight allocation in the third collection period only 8

Table S8. Parameters of the path model 9

Table S9. Model results of sex ratio per site. 10

Figures **11**

Figure S1. Overview of the 20 study sites in the surroundings of Freiburg, Germany. 11

Figure S2. Representative images of bees transporting (a) pollen on its scopa and (b) clay in its mandibles as nesting materials 12

Figure S3. Visualization of model results (only predictors with $p < 0.05$) for the influence of sex and internode diameter on pollen weight 13

Tables

Table S1. Identified flowering herbaceous plant species on study sites, which are suitable pollen sources for *Osmia cornuta* (according to Westrich, 2019. Die Wildbienen Deutschlands (2nd). Stuttgart: Eugen Ulmer).

Common name
Bugle (*Ajuga reptans*)
Common bird's trefoil (*Lotus corniculatus*)
Common grape hyacinth (*Muscari neglectum*)
Meadow buttercup (*Ranunculus acris*)
Bulbous buttercup (*Ranunculus bulbosus*)
Common dandelion (*Taraxacum officinale*)
Tufted vetch (*Vicia cracca*)
Bush vetch (*Vicia sepium)*

Table S2. Identified bee-relevant trees/shrubs (Rosacea trees and *Salix* spp.) and their combined crown area on study sites. The total crown area was divided through total site area for the analyses (area within a 250 m radius around the trap nest).

Common name	Total crown area [m²]
Apple (*Malus domestica*)	92,555.0
Cherry (*Prunus avium / cerasus*)	56,941.9
Plum (*Prunus domestica*)	18,601.2
Willow (*Salix* spp.)	15,083.5
Pear (*Pyrus communis*)	10,538.6
Cherry plum (*Prunus cerasifera*)	6,356.8
Blackthorn (*Prunus spinosa*)	4,268.2
Peach (*Prunus persica*)	2,074.1
Hawthorn (*Crataegus spp.)*	1,437.1
Apricot (*Prunus armenica*)	1,289.2
Quince (*Cydonia oblonga*)	288.2
Medlar (*Mespilus germanica*)	74.1
Almond (*Prunus amygdalus*)	18.9

Table S3. Summary of environmental variables that describe the 20 study sites. The range, median and mean (± SD) of each variable are shown.

Habitat variable	Range	Median	Mean ± SD
Temperature (° C)	-4.6 – 35.6	10.8	11.7 ± 8.0
Seminatural habitat connectivity (%)	0.2 – 99.2	19.8	35.5 ± 34.2
Pollen provision trees (%)	0.1 – 31.1	2.5	6.0 ± 7.6
Flower cover (%)	0.0 – 0.5	0.2	0.2 ± 0.2

Table S4. Overview of hatched offspring, sex ratio per site, total number of flights (including pollen and clay collection flights) and environmental predictors that describe the 20 study sites.

Site	Males	Females	Sex ratio	Total flights	Flower cover (%)	Pollen provision trees (%)	Seminat. habitat connectivity (%)
01	0	2	1.00	235	0.15	10.6	74.33
02	12	11	0.48	221	0.17	0.4	9.11
03	6	13	0.68	554	0.39	2.5	49.09
04	26	10	0.28	73	0.05	7.5	65.47
05	5	10	0.67	185	0.04	0.9	12.65
06	4	12	0.75	109	0.00	1.0	5.08
07	11	7	0.39	203	0.34	0.1	0.19
08	50	38	0.43	1,008	0.20	11.7	91.59
09	7	7	0.50	107	0.34	9.2	19.79
10	6	11	0.65	107	0.02	7.9	15.9
11	7	6	0.46	293	0.04	5.4	27.61
12	11	13	0.54	227	0.18	0.9	8.8
13	4	7	0.64	58	0.20	8.4	25.99
14	4	0	0.00	162	0.04	0.5	84.68
15	6	9	0.60	193	0.54	1.8	99.15
16	9	7	0.44	131	0.01	1.8	6.04
17	6	7	0.54	93	0.14	31.1	8.54
18	0	0	-	-	0.00	0.0	0.4
19	0	0	-	193	0.03	2.6	31.81
20	0	0	-	-	0.02	2.4	63.08

Table S5. Model structure of all generalized linear (mixed-effects) models and linear mixed-effects models. Total pollen flights were log_{10}-transformed to improve normality and variance homogeneity. The random intercept for the sex allocation probability model was dropped as it did not explain any variance. Word "seminatural" was abbreviated with "seminat.".

Response variable	Predictor	Random intercept
Population scale		
**Sex ratio** (= proportion of females in a population)<br>**per site** (binomial)	Temperature<br>Seminat. habitat connectivity<br>Pollen provision trees<br>Flower cover<br>Log_{10}(Pollen flight frequency)	None
Individual scale		
**Sex allocation probability**<br>→ brood cells developed into females = 1, brood cells developed into males = 0<br>(binomial)	Temperature<br>Seminat. habitat connectivity<br>Pollen provision trees<br>Flower cover<br>Collection day<br>Internode diameter	None
**Pollen weight**<br>→ resource allocation<br>(linear)	Temperature<br>Seminat. habitat connectivity<br>Pollen provision trees<br>Flower cover<br>Collection day<br>Internode diameter<br>Sex (male or female)	Reed internode nested in site
**Pollen collection duration**<br>→ resource allocation efficiency<br>(negative binomial)	Temperature<br>Seminat. habitat connectivity<br>Pollen provision trees<br>Flower cover<br>Recording day	Reed internode nested in site
**Clay collection duration**<br>→ resource allocation efficiency<br>(negative binomial)	Temperature<br>Seminat. habitat connectivity<br>Pollen provision trees<br>Flower cover<br>Recording day	Reed internode nested in site

Table S6. Component models for the path model. Pollen flight frequency was log_{10}-transformed to improve normality and variance homogeneity. Word “seminatural” was abbreviated with “seminat.”. . All models were tested for multicollinearity via variance inflation factors (always < 3, i.e. collinearity unlikely).

Response variable	Fixed effect	Random intercept
**Sex ratio per collection day**<br>(linear with total individual number per collection period as weights)	Temperature<br>Seminat. habitat connectivity<br>Collection day	Site ID
**Pollen flight frequency**<br>(linear)	Temperature<br>Seminat. habitat connectivity<br>Pollen provision trees<br>Flower cover	Site ID
**Flower cover**<br>(linear)	Seminat. habitat connectivity	None
**Pollen provision trees**<br>(linear)	Seminat. habitat connectivity	None

Table S7. Model results of pollen weight allocation in the third collection period only. The estimates (± SE), z-/t-value and p-value are shown. Results did not change in comparison to the model with all collection periods included (Table 1, main manuscript). Word “seminatural” was abbreviated with “seminat.”.

Response	Fixed effect	Estimate ± SE	t-value	p-value
Pollen weight	**Sex (male or female)**	**144.165 ± 7.338**	**19.646**	**<0.001**
	**Internode diameter**	**14.548 ± 4.888**	**2.976**	**0.006**
	Temperature	-20.978 ± 10.636	-1.972	0.110
	Seminat. habitat connectivity	-10.129 ± 7.991	-1.268	0.260
	Pollen provision trees	-1.295 ± 5.990	-0.216	0.833
	Collection period	-1.920 ± 9.521	-0.202	0.850
	Flower cover	-1.278 ± 9.005	-0.142	0.892

Table S8. Parameters of the path model (Fisher's C = 15.4, p = 0.64). Paths with a p-value <0.05 are highlighted in bold. Pollen flight frequency was log$_{10}$-transformed to improve normality and variance homogeneity. The relationship between Collection day and Temperature was fitted as correlated error. Word "seminatural" was abbreviated with "seminat.".

Response	Fixed effect	Unstandardised estimates	Range-standardised estimates	Standard error	p-value
**Sex ratio**	**Collection day**	**-0.09**	**-0.39**	**0.033**	**0.02**
Sex ratio	Log$_{10}$(Pollen flight frequency)	0.03	0.12	0.036	0.48
Sex ratio	Temperature	0.01	0.06	0.037	0.72
Log$_{10}$(Pollen flight frequency)	Seminat. habitat connectivity	0.02	0.02	0.237	0.94
Log$_{10}$(Pollen flight frequency)	Flower cover	0.41	0.41	0.234	0.10
Log$_{10}$(Pollen flight frequency)	Pollen provision trees	-0.07	-0.07	0.228	0.77
Log$_{10}$(Pollen flight frequency)	Temperature	-0.01	-0.01	0.144	0.97
**Flower cover**	**Seminat. habitat connectivity**	**0.38**	**0.38**	**0.175**	**0.04**
Pollen provision trees	Seminat. habitat connectivity	0.04	0.04	0.189	0.84
**Collection day ~**	**~ Temperature**	**0.63**	**0.63**	**-**	**<0.01**

Table S9. Model results of sex ratio per site. The estimates (± SE), z-/t-value and p-value are shown. No variable was significant (all p > 0.05). Word "seminatural" was abbreviated with "seminat.".

Response	Fixed effect	Estimate ± SE	z-/t-value	p-value
Sex ratio per site	Seminat. habitat connectivity	-0.267 ± 0.136	-1.954	0.051
	Flower cover	0.185 ± 0.137	1.352	0.176
	Temperature	0.155 ± 0.141	1.098	0.272
	Log_{10}(Pollen flight frequency)	0.075 ± 0.118	0.636	0.525
	Pollen provision trees	0.057 ± 0.141	0.400	0.689

Figures

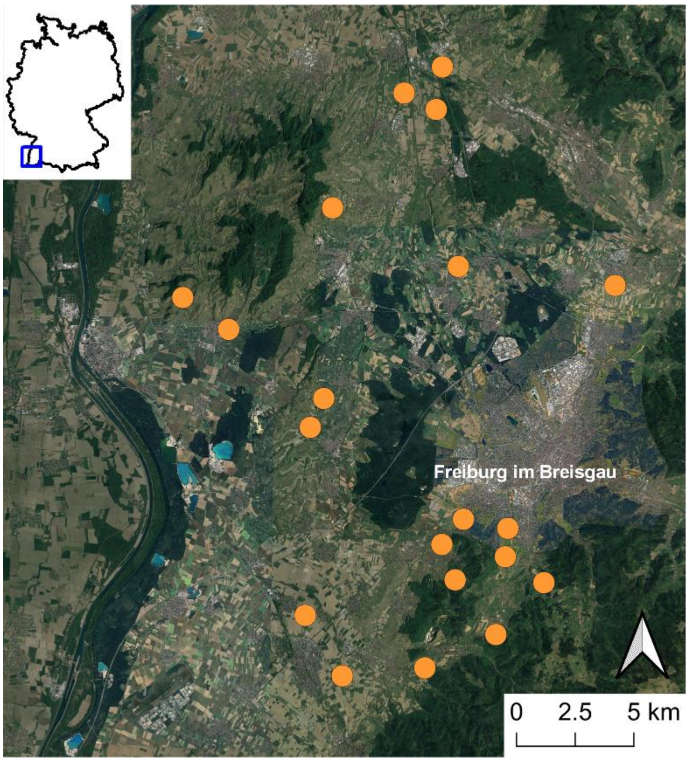

FIGURE S1. Overview of the 20 study sites in the surroundings of Freiburg, Germany. Minimum distance between two adjacent sites was 1.4 km. The study sites comprised of orchards, orchard meadows, extensively and intensively managed meadows, cropland and pastures.

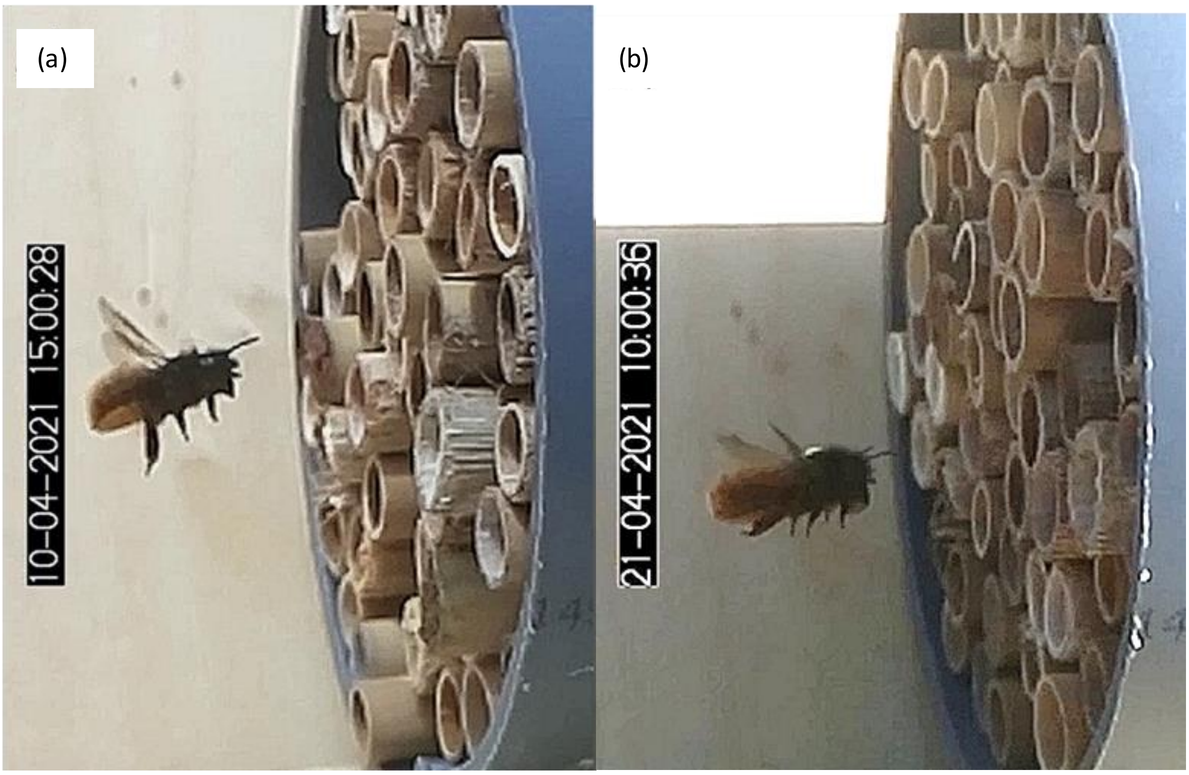

FIGURE S2. Representative images of bees transporting (a) pollen on its scopa and (b) clay in its mandibles as nesting materials (brightness was slightly increased). Pictures were extracted from video recordings that were obtained via the installed camera system during the observation period.

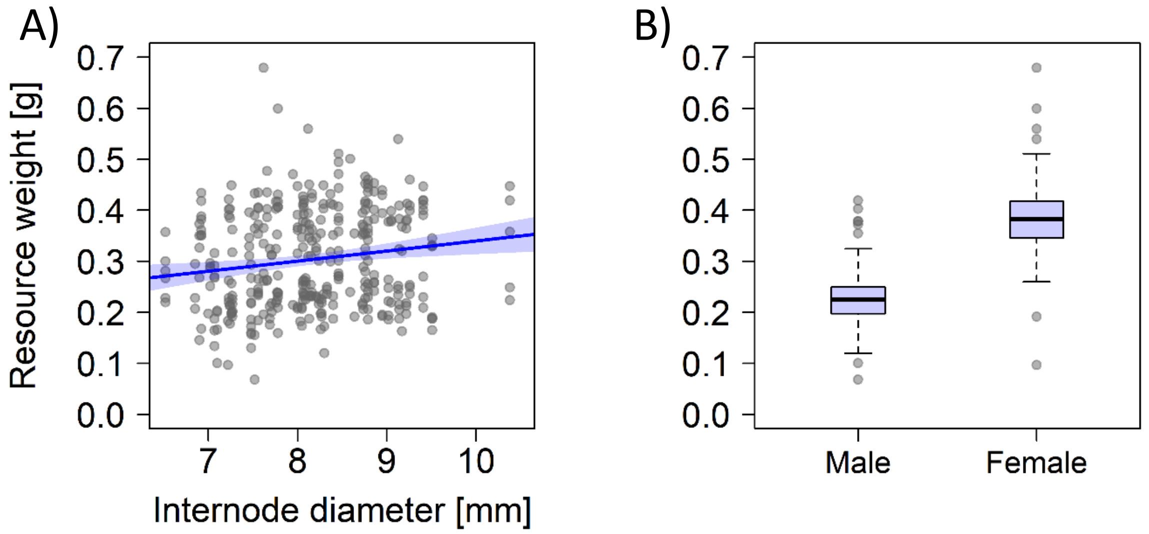

Fig. S3. Visualization of model results (only predictors with $p < 0.05$) for the influence of sex and internode diameter on pollen weight. More resources were provisioned with A) increasing diameter ($t = 2.976$, $p = 0.006$) and B) for female offspring ($t = 19.646$, $p < 0.001$). Solid lines indicate model predictions with 95% confidence intervals, which are depicted as shaded polygons. Please note that the graph uses resource weight in g for an easier interpretation. Statistical analyses were conducted in mg.
